# Advanced identification of global bioactivity hotspots via screening of the metabolic fingerprint of entire ecosystems

**DOI:** 10.1038/s41598-020-57709-0

**Published:** 2020-01-28

**Authors:** Constanze Mueller, Stephan Kremb, Michael Gonsior, Ruth Brack-Werner, Christian R. Voolstra, Philippe Schmitt-Kopplin

**Affiliations:** 1 Research Unit Analytical BioGeoChemistry, Helmholtz Zentrum München, German Research Center for Environmental Health, Neuherberg, D-85764 Germany; 2 NYUAD Center for Genomics and Systems Biology, New York University Abu Dhabi, Abu Dhabi, United Arab Emirates; 3 University of Maryland Center of Environmental Science, Chesapeake Biological Laboratory, Salomons, MD 20688 USA; 4 Institute of Virology, Helmholtz Zentrum München, German Research Center for Environmental Health, Neuherberg, D-85764 Germany; 5 Red Sea Research Center, Division of Biological and Environmental Science and Engineering (BESE), King Abdullah University of Science and Technology (KAUST), Thuwal, 23955-6900 Saudi Arabia; 6 Department of Biology, University of Konstanz, Konstanz, 78457 Germany; 7 Chair of Analytical Food Chemistry, Technische Universität München, Freising-Weihenstephan, D- 85354 Germany

**Keywords:** Drug screening, Biogeochemistry, Analytical chemistry

## Abstract

Natural products (NP) are a valuable drug resource. However, NP-inspired drug leads are declining, among other reasons due to high re-discovery rates. We developed a conceptual framework using the metabolic fingerprint of entire ecosystems (MeE) to facilitate the discovery of global bioactivity hotspots. We assessed the MeE of 305 sites of diverse aquatic ecosystems, worldwide. All samples were tested for *antiviral* effects against the human immunodeficiency virus (HIV), followed by a comprehensive screening for cell-modulatory activity by High-Content Screening (HCS). We discovered a very strong HIV-1 inhibition mainly in samples taken from fjords with a strong terrestrial input. Multivariate data integration demonstrated an association of a set of polyphenols with specific biological alterations (endoplasmic reticulum, lysosomes, and NFkB) caused by these samples. Moreover, we found strong HIV-1 inhibition in one unrelated oceanic sample closely matching to HIV-1-inhibitory drugs on a cytological and a chemical level. Taken together, we demonstrate that even without physical purification, a sophisticated strategy of differential filtering, correlation analysis, and multivariate statistics can be employed to guide chemical analysis, to improve de-replication, and to identify ecosystems with promising characteristics as sources for NP discovery.

## Introduction

Natural products (NP) are an attractive starting point for drug screening and discovery due to their high structural diversity and complexity^1–3^. Approximately 60% of all currently registered drugs originate from natural sources, which is particularly true for anti-infectives and cytostatic drugs^1–4^. However, it is becoming increasingly clear that the discovery rate of novel structures from traditional sources of NP is declining^5^. In this context, new original sources of NP can help in providing novel molecular entities for the development of lead compounds and to avoid increasing redundancy and rediscovery rates^6,7^.

In this study, we explored the potential of complex mixtures extracted from different aquatic ecosystems worldwide as novel sources for bioactive compounds. We focused our efforts on complex mixtures collected in aquatic ecosystems as molecules are being leached out of surrounding ecosystems and are being transported into water reservoirs by boundless water cycles (Fig. S1a). Considering the fact that living biota releases signaling or defense molecules into their adjoining environments^8,9^, it is only reasonable that traces of such bioactive molecules can be found in these samples. In general, the complex extracts used in this study contain intact molecules exuded, excreted, leached and otherwise released from any living and decaying biota within the ecosystem^10^. Their chemical richness is further increased by chemical and biochemical transformation and alteration processes^10^. In consequence and by extension of the definition of the metabolome (entirety of small molecules (<1 kDa) within an organism), these complex extracts can be considered as the metabolome of the entire ecosystem (MeE). Importantly, the sampling procedure of the MeE does not involve ecological impact. Hence, it can easily be applied as pilot screening for drug discovery and creation of a global natural product catalogue.

Due to the high complexity of the MeE (several thousand molecules per sample), in-depth chemical profiling requires ultra-high resolving analytical tools with a very high sensitivity and dynamic range^11,12^. In terms of mass spectrometry, such properties are currently only provided by Fourier transform ion cyclotron resonance mass spectrometers (FT-ICR-MS). Ultra-high resolution (UHR) thereby enables to distinguish between even very closely located m/z (mass/charge) signals. The extraordinary dynamic range poses the advantage of detection of compounds of very different concentrations and ionization efficiencies. Such analytical performance allows a *non-targeted (unbiased) chemical* analysis led by statistical modeling.

In terms of bioactivity screening, most conventional studies on effects caused by small molecules and NP have focused either on single molecular targets or general toxicity (*targeted biological analysis*)^13^. Unfortunately, the full bioactivity potential might not be detectable using these approaches. Imaging-based High-Content Screening (HCS) using a set of fluorescence dyes or antibodies that target several cellular structures has recently emerged as a promising tool for more holistic screenings^14–19^. It provides a powerful strategy for *non-targeted (unbiased) biological profiling* of alterations caused by small molecules and for prediction of compound-related mode of action (MoA)^14,15^. However, combined analyses of the in-depth chemical composition with comprehensive biological activity profiles are not reported in literature. To our best knowledge only Kurita *et al*.^20^ and Kremb *et al*.^21^ initiated this type of analysis, but with samples of low complexity and using only a limited set of cellular structures. Another strategy for the identification of NP candidates with promise is the screening for *antiviral* activities, such as inhibition of the human immunodeficiency virus type-1 (HIV-1). This has been successfully demonstrated for a broad variety of complex mixtures of natural products, using a robust phenotypic screening assay encompassing the entire HIV replication cycle (EASY-HIT)^22–26^.

We here present a global survey of combined chemical and biological profiling of divers MeE to pinpoint environments that should serve as promising starting points in future NP discovery studies. We performed in-depth characterization of the chemical composition of each MeE combined with (i) a well-established *cell-based antiviral* assay (EASY HIT anti-HIV-1) and (ii) with a *comprehensive* High-content Screening, which yields insights into the altered cell physiology of treated mammalian cells. For both assays, we employed diverse MS-based informatics approaches, including multivariate statistics and UHR molecular networking, to link the chemical composition with the obtained bioactivity of the sample.

## Results

### Chemical characterization of worldwide sampled metabolic fingerprints of entire ecosystems (MeE)

**Figure 1:**
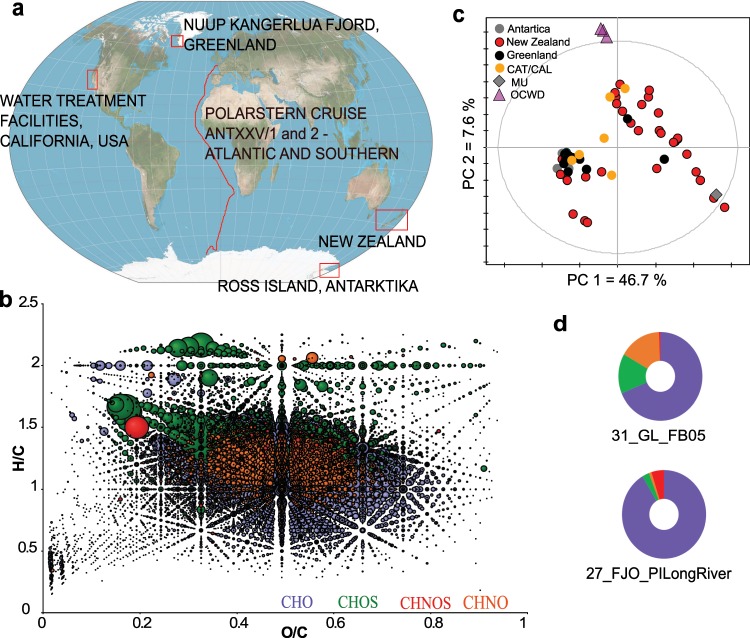
Sampling sites and their chemical characterization. An overview of the geographic origin of the analyzed MeE is indicated in the world map (map from Wikipedia, reuse permitted under the Creative Commons Attribution-ShareAlike 3.0 Unported license (CC-BY-SA 3.0, https://creativecommons.org/licenses/by-sa/3.0/), created by Strebe, https://en.wikipedia.org/wiki/World_map#/media/File:Winkel_triple_projection_SW.jpg, modified) (**a**). All samples were screened for their chemical composition via FT-ICR-MS analysis, which resulted in chemical fingerprints consisting out of several thousand m/z features per sample and their relative intensities. (**b**) The combined van Krevelen diagram of all MeE depicts a broad distribution across the chemical space of all elemental compositions (CHO (blue), CHOS (green), CHNO (orange) and CHNOS (red). Bubble sizes represent the summed detected intensities). **(c)** PCA score plot based on chemical fingerprints, in which samples are colored according to the geographic location of sampling gives an overview of similarities and differences within the samples’ organic content. **(d)** The chemical space at different sampling sites varied thereby profoundly, exemplarily shown for 31_GL_FB05 (taken in Greenland) and 27_FJO_PILong River (taken in New Zealand). These two samples were chosen as they pose a high *antiviral* activity, which is later described in detail. The ring charts give the quantitative distribution of elemental compositions (color coding according to **b**).

We applied *non-targeted* UHR mass spectrometry to capture the chemical space of 305 MeE samples collected in five continents (Europe, Africa, Australia, North America and Antarctica) at different sites in aquatic ecosystems (Fig. 1a). We included field samples of coastal and marine ecosystems, as well as along vertical and horizontal gradients of several fjords, which link terrestrial and marine ecosystems. The organic material contained in the water samples was concentrated by solid phase extraction (SPE) prior to analysis (Fig. S1b). SPE preparation of the samples furthermore ensured enrichment of molecules in a typical drug hydrophilicity range (logP of approximately −0.4 to +5.6). Each single sample yielded a distinct chemical fingerprint, consisting out of several thousand detected m/z features and their relative intensities (in total >31,000 different m/z features, Fig. S2). These fingerprints varied between the samples according to the sampling sites and reflect the geo-ecological origin of the samples. The captured chemical space is very broad and the detected m/z features are distributed in all compound classes (Fig. 1b), but with profound differences according to the sampling site of the MeE (Fig. 1c,d).

### Detection of *antiviral* activity in MeE and characteristics of related ecosystems

**Figure 2:**
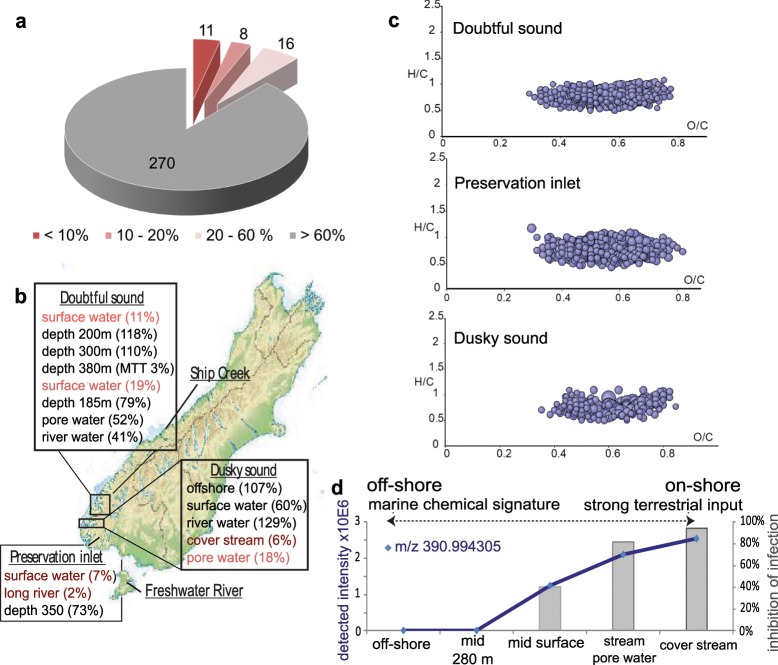
Summary of anti-HIV activity of MeE and related m/z features. All samples were tested for their HIV-1-inhibitory potency. A general overview of obtained results is given in the pie chart **(a)** (full data can be found in Table S1). We categorized the obtained viral inhibition in 4 groups: less than 10%, 10–20%, 20–60% of infection compared to non-treated infected cells. The number of samples belonging to each group is illustrated. Nine out of eleven samples with very high anti-HIV activity (<10% of infection) originate from fjords in New Zealand (map from Wikipedia, reuse permitted under the Creative Commons Attribution-ShareAlike 3.0 Unported license (CC-BY-SA 3.0, https://creativecommons.org/licenses/by-sa/3.0/), https://en.wikipedia.org/wiki/File:New_Zealand_relief_map.jpg, modified). **(b)** We observed a relation of sampling sites within the fjords and HIV inhibition: for all fjords only surface waters and waters with strong terrestrial input show *antiviral* activity (HIV infection is given in % in brackets; *antiviral* samples are highlighted red). In contrast, in waters taken at the same location but from deeper layers the *antiviral* activity was absent. **(c)** A correlation analysis of the detected intensity of m/z features and detected HIV inhibition was performed to extract putatively associated m/z features out of the dataset for each sampling site. For all fjords, a set of CHO containing molecules delivered correlation coefficients >0.8. All of these cover the region of polyphenols in the van Krevelen diagram. **(d)** Following the correlation analysis between presence of m/z features and observed HIV inhibition over different depths at one sampling site, we surveyed the presence of these anti-HIV-associated m/z features along the fjords. This is exemplarily shown for one representative m/z 390.994305. The detected intensity of this m/z in complex extracts sampled along the fjord is illustrated, as well as the detected HIV inhibition of the total extract. The concentration of the molecule is higher deep inside the fjords, while it is absent at the mouth of the fjords and in related off-shore samples.

At first, we tested the idea of using the MeE for screening for bioactivity hotspots with an *antiviral* assay. We tested all 305 samples using a cell-based full-replication assay capturing all steps of the HIV-1 life cycle^26^. The fluorescence-based assay uses adherently growing HIV-susceptible cells, with a stable fluorescent reporter gene activated by HIV Tat and Rev. Furthermore, a MTT assay was used to address cell viability upon treatment^26^. Results from virus inhibition assay and MTT tests are given in Table S1. Initially, 88% of the samples showed no effect on HIV infection, 5% (n = 16) resulted in a moderate inhibition (20–60% of infected cells compared with untreated cultures), 3% (n = 8) in a strong inhibition (10–20% infected cells) and 4% (n = 11) exhibited a very potent HIV-1 inhibition (less than 10% infected cells) (Fig. 2a). Only one sample showed a significant reduction of cell viability assessed by the MTT proliferation assay. This sample was collected in a deeper water layer (380 m) of the *Doubtful sound* Fjord (21_FJO_DS 380), New Zealand.

We connected the *antiviral* activity with the geo-ecological origin of the samples, and observed that virus inhibition was mainly present in MeE which were either sampled along fjords at specific sampling sites or collected off-shore in unrelated environments. Regarding the first mentioned point, activity was found for instance exclusively in surface waters along the *Doubtful sound* Fjord, while samples taken at the same location but in deeper layers (185 m or 300 m) exhibited no inhibitory effect on HIV-1 replication (Fig. 2b). Similarly, porewater from the organic horizon of forest soils and stream samples from *Dusky sound* also showed potent activity. Deep water samples from within the fjord were inactive, as was observed for other fjords, as well. The *Preservation inlet* surface water, as well as Long River (freshwater) sample discharging in the fjord showed strong anti-HIV activity, while the water collected deeper at 350 m was once again inactive. Due to these observations we reasoned that a terrestrial input is probably the source of the bioactive molecules. We used Pearson correlation to search for single m/z features or groups of m/z features which are associated with the observed anti-HIV activity of the complex extracts. Correlation coefficient calculations were done separately for each sampling site along the depth, and along the course towards the mouth of the fjord using the detected intensity of the single m/z feature and the observed antiviral potency of the complex extract. We focused on m/z features with a correlation coefficient >0.8. For all above described ecosystems a group of putative polyphenols were obtained, exemplarily illustrated for *Dusky sound*, *Doubtful sound* und *Preservation inlet* (Fig. 2c). These molecules show a declining abundance towards the mouth of the fjords (exemplarily illustrated for one m/z feature in Fig. 2d).

Interestingly, one sample fell in many ways from this above described series of antiviral samples containing a strong terrestrial input: the sample 31_GL_FB05. 31_GL_FB05 was clearly HIV-active and reduced the virus replication to five percent, but it originates from an off-shore marine ecosystem in Western Greenland, without any terrestrial input. The molecules in this sample that correlate with HIV inhibition contain nitrogen in addition to carbon, hydrogen and oxygen and occupy the van Krevelen region of H/C 1–1.5 and O/C 0.3–0.6 (not shown). Important to note is furthermore, that this sample harbors additionally many molecules, which have been detected in the other Greenland samples, too. Therefore, a narrow clustering of Greenland samples can be recognized in the PCA score plot (Fig. 1c).

Furthermore, we performed a supervised machine learning to capture m/z features with the highest statistical power for differentiation of anti-HIV active (excluding the marine sample 31_GL_FB05 as its chemical fingerprint is very different) and non-active samples. The Partial least square discriminant analysis ((PLS-DA), R² = 0.914, Q² = 0.687)) delivered a set of 10 m/z features as significantly enriched (>3 fold, p < 2.25*10E-5 Benjamini/Hochberg corrected for multiple testing) or exclusively present in the most potent *antiviral* active samples. Their molecular composition is given in Table 1. All molecules clustered closely together in the H/C and O/C ration of 0.5–1.2 and 0.4–0.6 and are uniquely composed of carbon, hydrogen and oxygen. We crosschecked NIAID (National Institute of Allergy and Infectious Diseases ChemDB: Division of AIDS Therapeutics Database), HMDB (Human Metabolome Database)^27,28^, KEGG (Kyoto Encyclopedia of Genes and Genomes)^29^ and LIPID MAPS Structure Database (LMSD)^30^ for related entries. None of the m/z feature is registered in the NIAID database, the biggest database for infectious diseases. One m/z feature corresponded to a Quercetin derivative (Quercetin 3-(2′-galloyl-alpha-L-arabinopyranoside)) within an error of 0.07 ppm in LMSD (Table S2). Moreover, we performed sustained off-resonance irradiation collision-induced dissociation (SORI-CID) of the most abundant m/z features out of these 10 for further structural information. The procedure allows precise isolation of m/z features and is the method of choice for complex samples. Ions of interest (parent ions) are isolated, radial accelerated and fragmented by collisions with argon atoms inside the ICR cell, which results in informative patterns of product ions. It mainly led to neutral losses of 18, 32, 44 and 76 (Table 2 SI), which belong to typical fragments such as H_2_O, CO_2_, CH_3_OH and a combination of CH_3_OH and CO_2_ (Table S3). The loss of H_2_O indicated the existence of hydroxyl functional groups whereas carboxyl groups were most likely present due to the loss of CO_2_^31^. Taking these fragmentation patterns together with the localization of the m/z features in the van Krevelen diagram (H/C 0.69–0.84 and O/C 0.52–0.62)^12^, the data strongly suggest the importance of highly oxidized polyphenols.

**Table 1:** Most important variables in projection.

m/z	Internal ID	ERROR [ppm]	H	C	O	N	S
343.04594	8069	0.017	11	17	8	0	0
527.08312	7109	−0.056	19	25	13	0	0
529.06238	8124	−0.084	17	24	14	0	0
529.09877	7061	−0.037	21	25	13	0	0
543.07803	7821	−0.063	19	25	14	0	0
559.07295	7115	−0.07	19	25	15	0	0
559.10933	2029	−0.062	23	26	14	0	0
571.07295	7826	−0.086	19	26	15	0	0
573.12498	7453	−0.043	25	27	14	0	0
585.0886	8395	−0.067	21	27	15	0	0

Common m/z features, which were extracted from the PLS-DA analysis as discriminat for HIV-inhibitory samples. Elemental composition [M-H]^−^, which were calculated using formulae calculator^55^, contain exclusively carbon, hydrogen and oxygen.

### In-depth phenotyping of *antiviral* MeE using HCS

**Figure 3:**
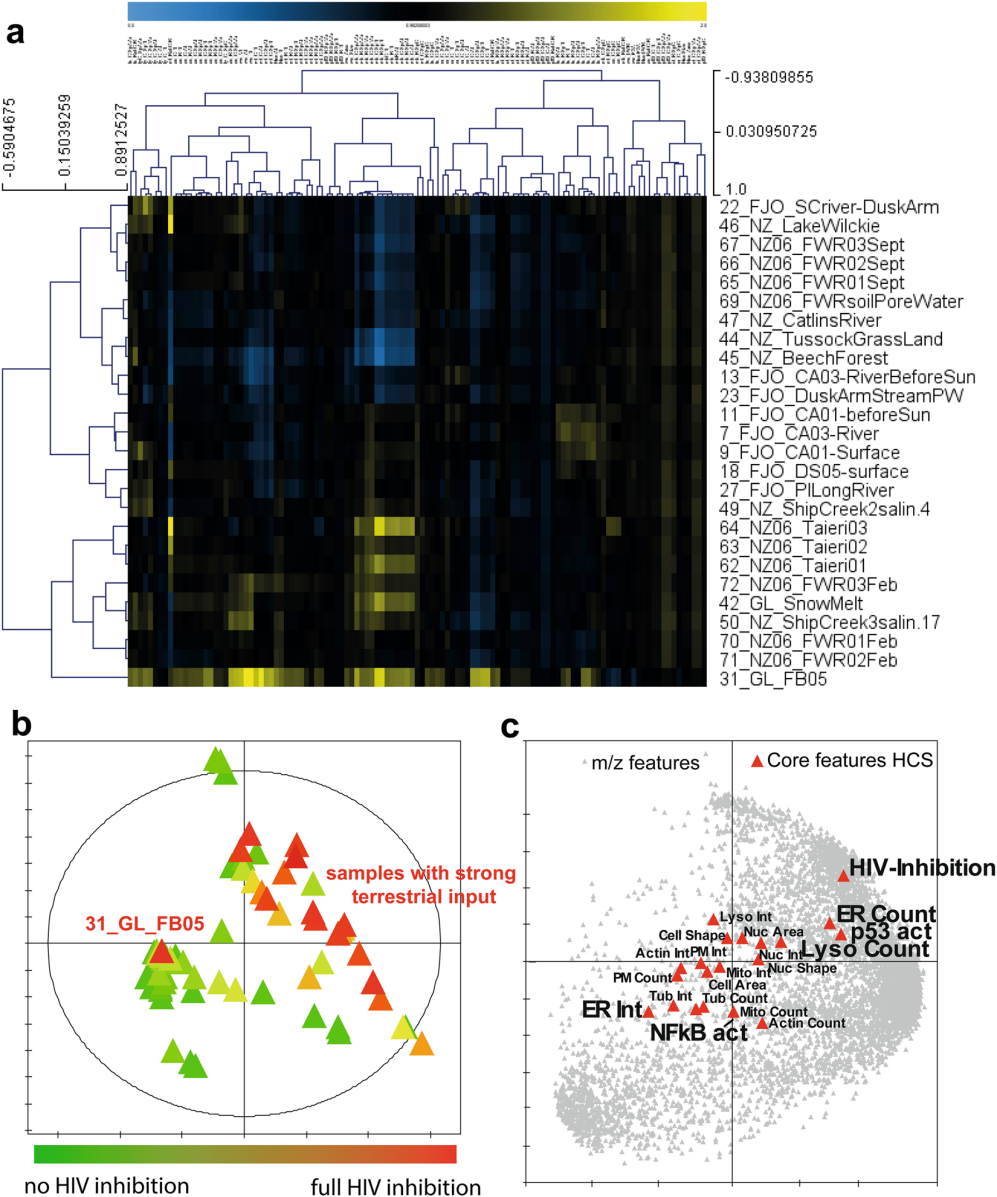
Obtained HCS results and integration with HIV inhibition and chemical fingerprints. Combined data analysis of the obtained HCS cytological profiles, anti-HIV-1 activity, and chemical fingerprints strongly suggests that the two main groups of *antiviral* MeE discovered in this study, cause virus inhibition through different MoAs and different sets of molecules. **(a)** Cluster analysis of all cytological profiles of *antiviral* samples (>50% inhibition) delivered two main clusters. Importantly, the open water sample 31_GL_FB05 shows a very different cytological profile compared to the other *antiviral* samples. (Colors indicate positive (yellow) or negative (blue) deviation from the mean of untreated control cells for each cellular feature (control = 1). Spearman rank correlation was used as a distance metric). **(b)** PCA score plot based on chemical fingerprints, in which samples are colored according to their HIV inhibition, show a close clustering of most *antiviral* samples in the first quadrant. These samples contain a strong terrestrial input and a related chemical fingerprint. Only one *antiviral* sample, 31_GL_FB05, does not belong in this cluster, which indicates that this sample contains a different set of molecules. **(c)** The PCA loading plot of combined data (HCS cytological profiles, HIV-1 inhibition, UHR mass spectrometric fingerprints) shows an overview of cellular core features, which are correlated/anti-correlated with the HIV inhibition and with a subset of m/z features. For easier readability the HCS parameters were limited to a set of core markers, which contains most of the information.

Following the assessment of antiviral activity of the complex extracts, a comprehensive High-content Screening (HCS) approach was used, which provides an unbiased hypothesis-free biological characterization of alterations caused by the complex extracts on a single-cell level. The HCS platform combines a set of fluorescents dyes targeting 11 cellular structures (nucleus, actin, tubulin, mitochondria, whole cell, endoplasmic reticulum, lysosomes, membranes, NFkB, Caspase 9, p53) resulting in 134 cellular measures and a characteristic cytological profile. This cytological profile covers subsequently a broad spectrum of cell physiology and can serve as a unique fingerprint for sample-induced or drug-induced alterations on mammalian cells^15^. Importantly, drugs that share a MoA deliver comparable cytological profiles, which allows the use of HCS as a prediction tool^14,15^. We screened ecosystems which were found to contain at least one antiviral sample. To cross-reference their cytological profiles to known MoA, we compared cytological profiles obtained from the MeE to a library of 720 single bioactive reference compounds selected from the LOPAC^®^1280 library of pharmacologically active compounds (Sigma Aldrich, international version, http://www.sigmaaldrich.com/life-science/cell-biology/bioactive-small-molecules/lopac1280-navigator.html)^15,32,33^. Detected cytological profiles of MeE taken at comparable sample sites within ecosystems are largely consistent (e.g. samples 67, 68, 69, NZ-FW; 62–64, NZ Taieri, Fig. S4). This indicates a common pool of bioactive molecules for these MeE. HIV-inhibitory samples yielded in general four major clusters with multiple sub-clusters by two-dimensional Spearman rank clustering (Fig. 3a). While the first three clusters are mainly a result of differently regulated membrane features, the last cluster show a high variation from the others over all cytological features. The last cluster consists exclusively out of the sample 31_GL_FB05. Therefore, at least two different MoA are inferential.

Non-targeted HCS, targeted anti-HIV screening results and chemical fingerprints were then integrated in an unsupervised statistical model to extract influenced cellular key parameters and m/z features. Importantly, the different types of data were combined through unit variance scaling and mean centering. A principal component analysis (PCA) score plot was generated, in which samples are colored according to their virus inhibition (Fig. 3b). Clustering between MeE correlated to the properties of the sampling site, with a lesser dependence on their geographical location. HIV-inhibitory samples cluster closely within the first quadrant, where samples taken in ecosystems with a strong terrestrial input are located. Only one *antiviral* active sample was plotted outside this cluster with this non-supervised procedure: The open water sample 31_GL_FB05 representing a marine ecosystem off-shore in Greenland.

The PCA loading plot (Fig. 3c) demonstrate a small number of cytological parameters, that are modified in cells treated with HIV-inhibitory mixtures. Core features of endoplasmic reticulum (ER), lysosomes, p53 and NFkB (nuclear factor kappa-light-chain-enhancer of activated B cells) are associated with HIV inhibition (Fig. S5a,b). A set of CHO containing compounds, highly likely to be polyphenols (Fig. S5c), followed this correlation for ER-, lysosomal, p53-related features. As an *in-silico* extraction of compounds correlating with NFkB expression over the entire dataset delivered only poor correlation coefficients, we focused here on a subset of samples with a clear effect on NFkB intensity (Fig. S5b). Plant-derived classes of compounds have been positively evaluated as inhibitors of the NFkB pathway^34^ and NFkB activation is known to be associated with HIV^34,35^. The correlation analysis between intensities of m/z features and NFkB total intensity over samples, which show a clear effect on NFkB, gave valid correlation coefficients (>0.8). The molecular composition of these compounds strongly suggest their polyphenolic nature (not shown).

**Figure 4:**
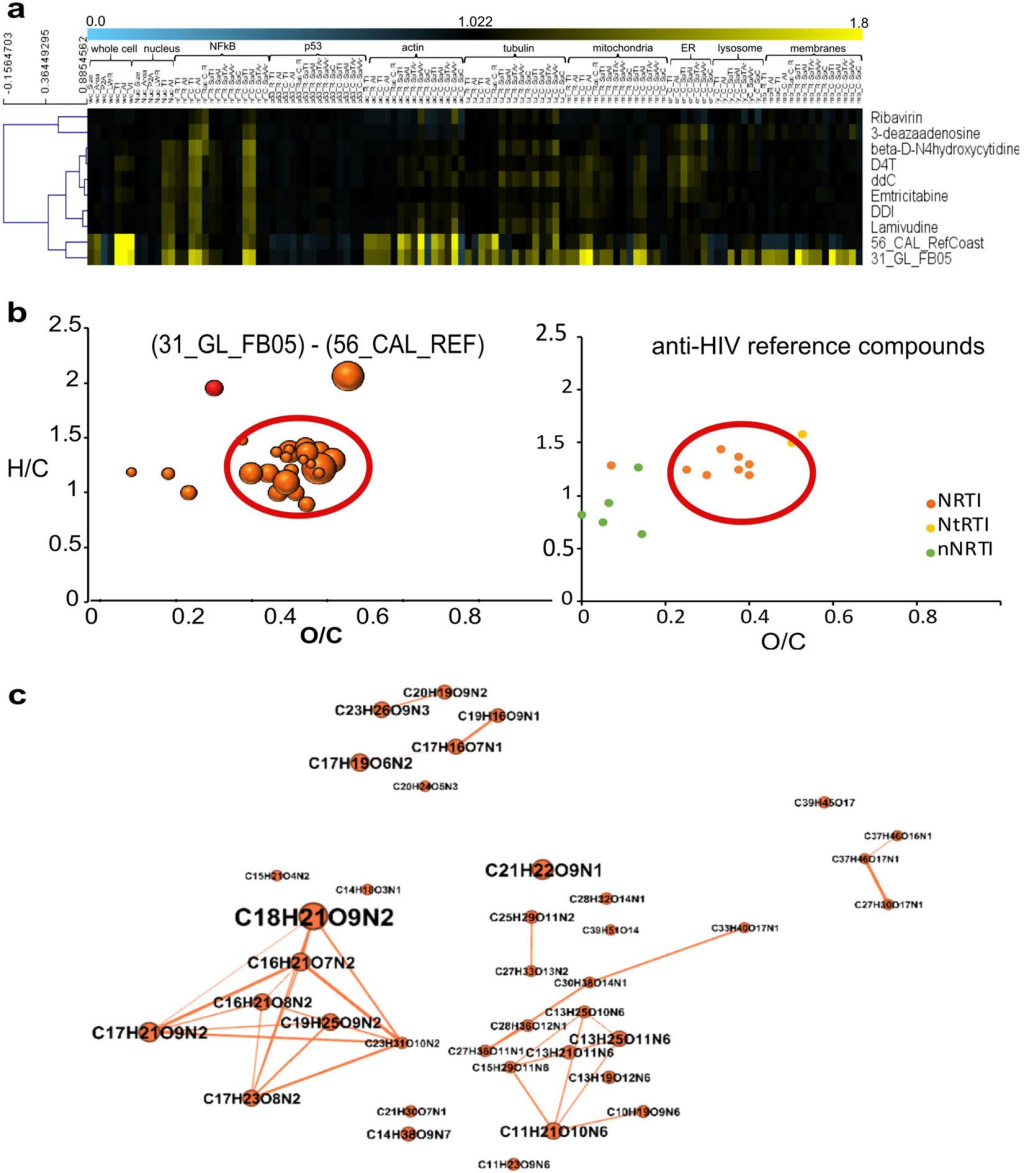
Detection of NRTI-like signatures in a marine *antiviral* MeE. The marine sample 31_GL_FB05 shows a close clustering to HIV reference compounds (NRTI). M/z features, which are uniquely present in this sample, cover the same chemical space as these NRTIs and show related elemental composition. **(a)** Secondary analysis integrating reference compounds and cytological profiles obtained from MeE delivers a close clustering of reference nucleosidic inhibitors of the HIV-1 RT with two MeE samples: the anti-HIV active sample 31_GL_FB05 und 56_CAL_ref coast. These two samples differ particularly in the membrane marker region, with the HIV-1 active sample showing strong positive deviation. Colors indicate positive (yellow) or negative (blue) deviation from the mean of untreated control cells for each cellular feature (control = 1). Spearman rank correlation was used as a distance metric. **(b)** M/z features were filtered for their presence in 31_GL_FB05 and absence in 56_CAL_RefCoast (which clusters with sample 31_GL_FB05 by cytological profiles but does not show HIV-1 inhibition). Filtered m/z features cover the same chemical space as NRTIs (CHNO (orange), CHNOS (red), bubble sizes indicate detected intensity, NRTIs - Nucleoside analog reverse-transcriptase inhibitors, NtRTIs - Nucleotide analog reverse-transcriptase inhibitors, nNRTIs - Non-nucleoside reverse-transcriptase inhibitors). **(c)** Network analysis of these m/z features delivered three molecular families given by their elemental compositions. All of the correlated molecules contain nitrogen.

The open water sample 31_GL_FB05 showed a highly unique cytological profile with broad activities across almost all observations (Fig. 3a). This particular sample also exhibited strong inhibition of HIV-1 replication. In the secondary analysis, we matched cytological profiles obtained from the samples to a library of more than 500 compounds with assigned MoA, including anti-HIV activity. The cytological profile of the potent *antiviral* MeE clustered closely to a set of FDA-approved nucleosidic HIV-1 reverse transcriptase inhibitors (NRTIs) pointing to the presence of molecules with anti-HIV-1 RT (reverse transcriptase) activity (Fig. 4). Interestingly, a second sample (56_CAL_RefCoast) matched closely with the same set of RT-inhibitory reference compounds (Fig. 4a). However, this sample did not exhibit *antiviral* activity in our assay. While both samples show high correlation across profiles, we found strong differences particularly in the membrane marker region, with the HIV-1 active sample showing strong positive deviation. We compared the chemical fingerprints of these two samples. The anti-HIV biologically active open water sample harbors a unique set of specific nitrogen containing molecules (Fig. 4b), which cover the same space as NRTIs in the van Krevelen diagram. A network analysis of the UHR m/z feature revealed a partial chemical relation of these compositions (Fig. 4c).

## Discussion

NP are indispensable to modern medicine^1–3^. Nevertheless, discovery rates of novel chemical entities from traditional sources of NP are decreasing^5^. We therefore propose the use of the MeE in pilot screenings for localization of biodiversity hotspots of bioactive small molecules. With this first proof of principle study we could successfully detect distinct biological activities of this type of samples. We were able to describe characteristics that pinpoint ecosystems with high HIV-inhibitory potential and successfully correlated series of molecules in these specific environments. Although physicochemical isolation of single molecules is theoretically possible, we focus here on a strategy to accelerate the search for bioactive NP hotspot-locations. To our knowledge this is the first instance of combining UHR mass spectrometry data with an *unbiased* HIV full replication assay and a comprehensive *unbiased hypothesis-free* cellular phenotyping approach for such complex mixtures.

We assessed the bioactivity potential of MeE using cell-based screening assays as they mimic relevant *in vivo* conditions and allow for the simultaneous testing of effects on multiple steps in complex biological systems^22,26,36^. At first, an a*ntiviral* testing was used to validate our working hypothesis. We observed two kinds of ecosystems with an *antiviral* activity. On the one hand, samples collected in fjords with strong terrestrial influence showed potent anti-HIV activities. The Fiordland National Park samples were extracted along several fjords, which are coastal ecosystems that are strongly influenced by freshwater. Extreme levels of annual precipitation and steep mountain ranges rising from sea level result in a rich terrestrial input^37^. Interestingly, New Zealands’ forests are largely comprised of endemic species, which is especially true for woody plants and which likely contributes to the unique composition of the organic matter found in the coastal water bodies around New Zealand^38^. Statistical tests highlighted a set of putative polyphenols potentially responsible for the antiviral effect. Additional support of a terrestrial origin of anti-HIV active substances were found in streams sampled around *Beech Forest* and *Ship Creek* and in *Lake Wilckie (East coast South Island, NZ)*, as well as along the *Freshwater River* draining lowland located on Stewart Island. HIV inhibition was observed to be inversely correlated with increasing salinity^37^, which again demonstrates a mainly terrestrial origin of active substances, rather than a marine source. On the other hand, an unrelated marine sample collected off-shore in Greenland showed a strong virus inhibition. Here, a different set of molecules correlated with HIV inhibition.

We then expanded our study with a comprehensive phenotypic profiling. *Non-targeted* imaging-based HCS yielded for the first time detailed insights into bioactivity profiles of such complex natural material. High-content Screening is still a relatively new technology, especially regarding the combination of several dyes. It can be applied to both, single molecules as well as complex mixtures of molecules^14,15,18,20,39^. HCS provides rich information on multiple levels of cell physiology^40^ and is able to predict compound-related MoA by comparing cellular phenotypes characterized by cytological profiles to collections of molecules with known effects on multiple target classes^32,33^. We intended to address two goals with this additional screening step: (i) information regarding the putative MoA and (ii) expansion of our analytical approach from a combination of *targeted antiviral*/*non-targeted* chemical profiling towards *non-targeted*/*non-targeted* profiling. For both points we got positive results. The detected cytological profiles followed again a clear geo-ecological trend. On the one hand, we detected largely consistent cytological profiles for intriguing samples with a strong terrestrial input, as it is the case after snow melting or deep inside fjords. For those, the correlation of *antiviral* activity with multifactorial high-resolution phenotypic profiles allowed for the detection of multiple cellular features (like ER, lysosomes, and NFkB pathway) specifically affected. A positive correlation of lysosomal induction (increased lysosomal spot area) with HIV-1 inhibition was apparent, especially in the samples with highest *antiviral* activity. It is likely that increased lysosomal activity eventually leads to the degradation of viral components and thereby diminishing productive viral infection, as inhibition of lysosomal activity might result in an increase of HIV infectivity via the endocytic pathway^41^. We clearly observed polyphenols to be characteristic for samples that induced lysosomal effects. This is in agreement with the previous finding that the protective effects of polyphenols are in part due to a modulation of the lysosomal catabolic process in a concentration-dependent manner^42^. Furthermore, we found a negative correlation of two ER-related features (ER spot area and ER total Intensity, Fig. S5a,b) with *antiviral* activity. The ER plays an important role in the HIV-1 secretory pathway and it is possible that compounds targeting this step in the HIV-1 replication cycle lead to reduced HIV-1 infectivity. Moreover, we found a negative correlation of NFkB total intensity with *antiviral* activity. It is well known that HIV-1 gene expression is stimulated by NFkB binding to LTR elements and that, in turn, reduced levels of NFkB decreases HIV-1 proviral expression^35^. On the other hand, we found a marine sample that showed a strong correlation with cytological profiles of therapeutically used NRTIs. Here, a set of nitrogen containing molecules was differentially filtered and might reflect an algal bloom at the Greenland off-shore site. Notably, the differentially filtered nitrogen containing molecules occupy the same chemical space as NRTIs.

## Conclusions

In the presented proof-of-concept study we assessed functional and chemical aspects of complex mixtures from various ecosystems worldwide on a single cell level and in an omics scale. We illustrate that even without physical isolation of unique compounds^43–47^ a sophisticated strategy of differential filtering, correlation analysis and multivariate statistics can be used to guide chemical analysis and sample prioritization. We showed for the first time that natural complex samples simply taken by water collection in ecosystems harbor distinct biological activities and can be used to catalogue NP worldwide. As sampling of these kind of mixtures does not harm the ecosystem we suggest to use them for pilot screening to identify most productive sites for future novel NP efforts. Furthermore, we believe that the promising combination of technologies of *non-targeted* biological and *non-targeted* chemical analysis will strongly accelerate future bioactivity studies.

## Methods

### Sample material

In this study, we selected complex geochemical mixtures of various nature (marine, terrestrial, man-altered) and with a high diversity in their properties.

The sampling area and optical properties of water samples collected in *Fiordland National Park (NZ)* was previously described in detail^37,48^. Samples were collected at various depths within *Doubtful sound*, *Dusky sound* and *Preservation inlet*. Sampling was undertaken in Austral winter, but the low salinity layer (LSL) was still present which demonstrates the high amount of freshwater transported into the fjords, even under unstable stratification conditions. Photochemical effects were likely to be very minimal and the collected material was mainly subject to microbial transformations. Surface fjord waters started to freeze over at some places and water temperature ranged from 0 °C at the surface to 12 °C at depth in the fjords. Dissolved organic matter (DOM) in New Zealand fjords is derived from pristine low elevation evergreen temperate rain forests and higher elevation evergreen beech forests. The production of organic matter, leached out from soils by high rainfall volumes, is enormous and strongly colors the LSL that sits on top of clear ocean waters^37,48^.

Further samples were collected in the *Catlins region (NZ)* and the *Catlin River (NZ)*. This river drains a low elevation temperate rainforest on the East Coast of the South Island of New Zealand. *Lake Wilkie* is also located in the Catlins and is a coastal highly colored small lake dominated by sphagnum moss species. Samples from the low elevation temperate rainforest were collected on the *West Coast of the South Island (NZ)* in the so-called *Ship Creek*, where freshwater is mixing quickly with seawater in this very small estuary, and on *Stewart Island* along a salinity gradient in the Freshwater River. To be able to constrain higher elevation sources of DOM, we also included samples from a stream draining *Tussock grassland* and from an evergreen beech forest also located on the South Island of New Zealand.

Additionally, samples were collected during the expedition ANTXXV/1 and 2 of R/V *Polarstern* along a *transect in the East Atlantic Ocean and the Atlantic sector of the Southern Ocean*. Samples of surface and deeper waters were filtered and both, the water bodies as well as the filters were tested for their HIV-1-inhibitory potential. Details on the samples are given in Schmitt-Kopplin *et al*.^49^ and Ksionzek *et al*.^50^.

A third region of sampling was located in *Antarctica*. Samples were collected from underneath the shelf ice of Ross Island.

Furthermore, sampling was done along the *Nuup Kangerlua Fjord, Greenland*^51^.

A last set of samples is of different nature. We included DOM of secondary treated effluent: (i) from the Orange County Sanitation District after it had passed through the microfilteration process of the advanced water purification facility and indirect water reuse facility at Orange County Water District (OCWD)^51^; (ii) from a deep aquifer raw water that is used as source water to the Mesa Utilities drinking water treatment plant located in Costa Mesa, California; and (iii) from a sewage leakage on Catalina Island, California (CAT/CAL).

### Sample preparation

Samples were directly prepared at the sampling sites and thereafter stored on ice or at −20 °C until analysis. Sampling procedures are described in previous publications^37,48–51^. All samples were filtered using Whatman GF/F glass fiber filters, acidified to pH 2 with high purity hydrochloric acid and then extracted/enriched by SPE according to previously published protocols^52,53^. SPE was performed using Agilent Bond Elut PPL cartridges containing a functionalized styrene-divinylbenzene polymer. Following the standard operation procedure, the cartridge volume was adapted according to the sample volume and according to the DOM concentration (5 g or 1 g PPL resin) to achieve a maximum concentration of organic material^53^.

### Ultrahigh resolution mass spectrometry and raw data processing

Ultrahigh-resolution mass spectra were acquired on a Bruker solariX ion cyclotron resonance Fourier transform MS (Bremen, Germany) equipped with a 12 Tesla superconducting magnet and an Apollo II source in negative electrospray ionization mode. Samples were injected with a constant flow rate of 120 µl/h, nebulizer gas pressure of 2.2 bar and drying gas pressure of 4 bar at 200 °C. Accumulations time was 0.4 sec. The applied ESI voltage were 3600 V capillary voltage and −500 V end plate offset. The spectra were acquired using a time transient of 4 MW. MS parameters were optimized to reach a maximum of sensitivity in the m/z range 120–800. Transfer optic parameters were therefore ToF 0.6 msec, frequency 4 MHz and RF amplitude of 175 Vpp. 500 scans were acquired for each sample.

An injection of the reference material from the International Humic Substance Society (IHSS) Suwanne River Fulvic Acid was used for quality control at the beginning of each batch analysis. Spectra were externally calibrated first on clusters of arginine (2.5 mg/l in methanol) and internally calibrated on a fatty acid reference list in negative mode; calibration errors in the relevant m/z range were always below 100 ppb. The spectra were exported from Data Analysis using a Signal/Noise (S/N) minimum of 2 and aligned through an in-house written software using a maximum discrepancy of 1 ppm; the data were stored in a matrix. Missing values were imputed with random variables, reflecting minimal values in the data set. Therefore, a vector was built, which contain the minimum detected intensity for each sample over the entire dataset. Missing values were afterwards imputed by randomly generated integers included in this vector (Excel, 2016, Microsoft). Signal intensities were normalized to the sum of intensities of all detected m/z features per sample^54^.

Elemental compositions of detected m/z feature were calculated using the in-house written formulae calculator considering H, C, N, O and S (maximal error <0.2 ppm)^55^. Elemental compositions were filtered according to the seven golden rules, which consider restrictions for the number of elements, LEWIS and SENIOR chemical rules, isotopic patterns, hydrogen/carbon ratios, elemental ratio of nitrogen, oxygen, phosphor, and sulphur versus carbon, element ratio probabilities and presence of trimethylsilylated compounds^56^. Single charged m/z features with at least one isotopic feature were considered valid. Data from all analyzed samples were visualized in van Krevelen diagrams, in which hydrogen-to-carbon (H/C) atomic ratios (y-axis) against oxygen-to-carbon (O/C) atomic ratios (x-axis) are plotted. Hereby, H/C ratio reflects relative aliphaticity and aromaticity (double bond equivalents – DBE), whereas the O/C ratio relates to oxygenation (oxidation and reduction processes)^12^. The benefit of such visualization is an easier and faster interpretability of complex mass spectra as areas of certain compound classes can be defined^12^. Even though attention has to be paid, that this interpretation is not a chemical identification. Detected intensities are represented by bubble sizes.

Database search was done in MassTRIX allowing negatively charged masses (M-H)^−^ and a maximal ppm error of 1 ppm as only restrictions (http://masstrix3.helmholtz-muenchen.de/masstrix3/^57,58^) and a NIAID (National Institute of Allergy and Infectious Diseases ChemDB: Division of AIDS Therapeutics Database, http://chemdb.niaid.nih.gov) survey was performed manually for all important elemental compositions.

### Fragmentation experiments of relevant m/z features

M/z features which were under the top 100 VIPs and which showed at least a fold change of 3 difference between HIV active and non-active samples were selected for fragmentation experiments (n = 10). SORI-CID (sustained off-resonance irradiation collision-induced dissociation) was performed in negative electrospray mode. Mass spectra consisting of 20 scans were acquired on parent and fragment ions (Table S3). SORI power and isolation power were adapted according to requirements of the m/z feature.

### Cell culture

HEK293T (for virus stock preparation), LC5-RIC (for EASY HIT assay) and HeLa cells (for HCS) and their culture are described in^15,26^. Briefly, HEK293T cells were seeded into 6-well plates at 10^5^ cells in 2 ml of cell very-low-endotoxin (VLE)-RPMI 1640 medium (Biochrom AG, Berlin, Germany) supplemented with 10% fetal bovine serum (Biochrom AG) and 1% antibiotic-antimycotic solution (Gibco) before transfection. LC5-RIC cells contain a stably integrated reporter gene for expression of DsRed upon HIV-1 infection. The cells were cultured in cell culture flasks (T-185 Nunc Solo Flask, Nunc International, Wiesbaden, Germany) and maintained at 37 °C, 5% CO_2_ using DMEM (Gibco, Karlsruhe, Germany) culture medium supplemented with GlutaMAX^TM^-I (L-alanyl-L-glutamine), pyruvate and 4.5 g/l glucose (all from Gibco, Karlsruhe, Germany), 10% fetal bovine serum (FBS, Biochrom AG, Berlin, Germany), 1% antibiotic-antimycotic solution (Gibco, Karlsruhe, Germany), 1% sodium pyruvate (Gibco, Karlsruhe, Germany). In addition, 0.74 mg/ml geneticin (G418 sulfate; PAA Laboratories, Pasching, Austria) and 0.125 mg/ml Hygromycin B (PAA Laboratories, Paching, Austria) were added every second passage, to ensure stable expression of the CD4 receptor and general stability of the reporter construct. The cells were kept for a minimum of 48 h in medium without geneticin and hygromycin B prior to the experiments. HeLa cells (parental HeLa cell line, ATCC® CCL-2™) were seeded into 384-well plates at a density of 2,000 cells per well in a volume of 25 µl of Dulbecco’s modified Eagle medium (DMEM containing GlutaMAX-1; Life Technologies) containing 10% fetal bovine serum (Life Technologies) and 1% antibiotic-antimycotic solution (Life Technologies) and cultured under 37 °C and 5% CO_2_ for 24 h prior treatment with complex mixtures. The cells were used for a maximum of ten passages.

### EASY HIT- HIV full replication assay

A full description of the assay procedure and virus stock preparation used in this study is detailed in Kremb *et al*.^26^. In brief, 24 h after seeding the cells into microwell plates, the cell culture medium was removed and the sample (dissolved in 100 μl cell culture medium) and 20 μl virus inoculum were added to the cells. Samples were prepared by drying of 10 µl of the original solution and re-dissolving in 330 µl of cell culture medium. 100 µl sample was added per well. Experiments were performed in triplicates. Cells were incubated for 48 h after addition of sample and virus inoculum. Cell culture supernatants were then removed from the treatment plate and fluorescence intensities of each culture measured. To measure effects of treatment on production levels of infectious virus, 20 μL of culture supernatants from treatment plates were transferred to uninfected LC5-RIC cells seeded in 96-well plates, plates incubated for 72 h and fluorescent intensities measured. Fluorescent measurements were performed with with a Tecan infinite M200 (Tecan, Crailsheim, Germany) at the monochromator wavelengths of 552 nm for excitation and 596 for emission.

Subsequently, viability of LC5-RIC cells was determined via a MTT test (3-(4,5-dimethylthiazol-2-yl)- 2,5-diphenyl tetrazolium bromid) by incubation of cell cultures with 50 µg of MTT solution (Sigma-Aldrich, Taufkirchen, Germany) in 100 µl of cell culture medium for 2 h at 37 °C as described in^26^.

### High-content screening

High-content Screening of cellular alterations upon MeE-treatment was performed according to a published protocol^15^ targeting 11 cellular structures (Table 2) and resulting in unique cytological profiles of 134 features. Cells were treated with 25 µl of samples (re-dissolved in DMEM cell culture medium) in 4 replicates. After 24 h of treatment four different cell-staining protocols were applied (described in detail in Kremb *et al*.^15^). A Cellomics ArrayScan VTI (Thermo Fisher Scientific) platform equipped with a 10x objective (Zeiss Plan Neofluar, NA 0.3) was used. Images were analyzed using the Compartmental Analysis Bio Application (Cellomics, Thermo Fisher Scientific). A minimum of 500 valid objects were analyzed per well. Cell cycle analysis and analysis of cell loss were accomplished by using the Cell Cycle Bio Application (Cellomics, Thermo Fisher Scientific) using at least 2000 valid objects. Raw data from automated image analysis for each cytological feature were related to corresponding values from control wells where the control was set to 1. All cytological features of a given fraction or reference compounds (LOPAC^®^1280 – The Library of Pharmacologically Active Compounds, Sigma-Aldrich, Taufkirchen, Germany) were combined to result in a cytological profile.

**Table 2:** Overview of High-content Screening (HCS).

Panel	Cellular target	Description	Staining solutions
Panel 1	Nucleus	Nuclear parameter	Hoechst33342 (Thermo Fisher Scientific)
	Actin	Cytoskeleton	Phalloidin-FITC (Sigma Aldrich)
	Tubulin	Cytoskeleton	Beta-tubulin antibody and GAM-DyLight 550 (Thermo Fisher Scientific)
	Mitochondria	Mitochondrial membrane potential	MitoTracker® Orange CMTMRos (Life Technologies)
Panel 2	Whole cell	Whole cell morphology	Wheat Germ Agglutinin, Alexa Fluor® 488 Conjugate (Life Technologies)
	ER	Endoplasmic reticulum	ER-Tracker Blue-White DPX (Life Technologies)
	Lysosomes	Lysosomes	LysoTracker Red DND-99 (Life Technologies)
	Membrane	Membrane	Wheat Germ Agglutinin, Alexa Fluor® 488 Conjugate (Life Technologies)
	Nucleus	Nuclear parameters	Hoechst33342 (Thermo Fisher Scientific)
Panel 3	NF-ĸB	NF-ĸB activation/distribution	Antibody for NF-ĸB and GAR-DyLight 550 (Thermo Fisher Scientific)
	Nucleus	Nuclear parameters	Hoechst33342 (Thermo Fisher Scientific)
Panel 4	Caspase 9	Caspase 9 activation/distribution	Caspase 9 antibody and GAM-DyLight 550 (Thermo Fisher Scientific)
	p53	p53 activation/distribution	p53 antibody and GAR- DyLight 488 (Thermo Fisher Scientific)

Eleven cellular structures were examined by HCS, using 4 panels of of staining solutions with different fluorescent dyes and antibodies.

### Statistical analysis

Pearson correlation was used to screen for associated m/z features with the observed anti-HIV-1 activity of the complex extracts (Excel 2016, Microsoft, USA). Therefore, the correlation coefficient of the detected intensity and the observed antiviral potency of the complex extract was calculated for each single m/z feature. We considered correlation coefficients >0.8 as relevant. Principal Component Analysis (PCA) models have been used for data visualization and for discovery of natural occurring patterns as well as for identification of putative outliers. Mean centering in combination with unit variance scaling has been applied for the data of this study (SIMCA-P^©^ 9 (Umetrics, Umea, Sweden)). After exploration of naturally occurring patterns with unsupervised methods, the data has been further analyzed with Partial Least Square Discriminat Analysis (PLS-DA) in SIMCA-P^©^ 9 (Umetrics, Umea, Sweden). R² was observed to be 0.914. A seven-fold cross-validation has been applied, which resulted in a Q² of 0.682. Furthermore, a permutation test for evaluation of putative overfittings has been done with 200 permutations. The regression of the correlation coefficient between the original Y and the permuted Y versus the cumulative R^2^ and Q^2^ intercepts the x-axis at R² = 0.0887 and Q² = −0.125. Therefore, the model has met all the required quality criteria to be considered valid and not overfitting. M/z features with a VIP-VALUE (variable importance in projection) >1.98 have been considered relevant. Wilcoxon-Mann-Whitney test has been further used to assess the statistical significance of these m/z features (MultiExperimentViewer MeV v4.6.2^59^). P-values have been Benjamini/Hochberg corrected for multi testing. All cytological profiles were normalized to the average of cytological profiles detected for control cells and subjected to hierarchical clustering using complete linkage clustering with optimized gene leaf order and a Pearson correlation using Multi Experiment Viewer (MeV v4.9^59^, Dana-Farber Cancer Institute, Boston, MA, USA).

## Supplementary information

Supplementary Information.

## Data Availability

All data needed to evaluate the conclusions of the paper are present in the paper and/or the Supplementary Material. Additional data related to this paper may be requested from the authors.
