# Supplementary material for: Advanced identification of global bioactivity hotspots via screening of the metabolic fingerprint of entire ecosystems
Source: Sci Rep. 2020 Jan 28;10:1319. doi: 10.1038/s41598-020-57709-0 (PMC6987164; doi:10.1038/s41598-020-57709-0)
Supplement: Supplementary file 1 — Supplementary Information. [file 41598_2020_57709_MOESM1_ESM.docx]

Supplementary Materials for

***Advanced identification of bioactivity hotspots via screening of the metabolic fingerprint of entire ecosystems***

Constanze Mueller^1+*^, Stephan Kremb^+2,5^, Michael Gonsior ^3^, Ruth Brack-Werner^4^, Christian R. Voolstra^5,6^, Philippe Schmitt-Kopplin^1, 3, 7^*

Correspondence to: [*constanze.mueller@helmholtz-muenchen.de*](mailto:constanze.mueller@helmholtz-muenchen.de)


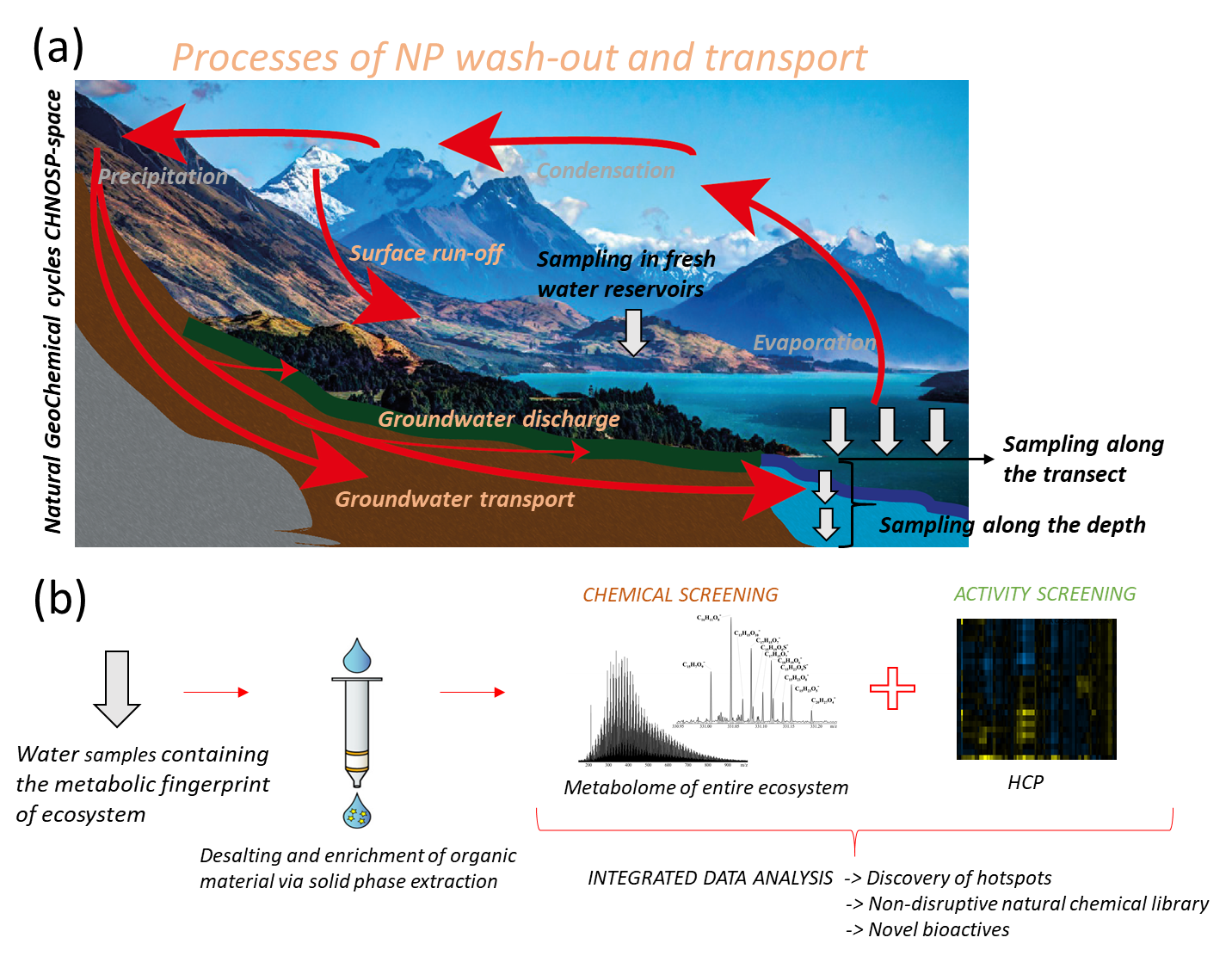


Fig. S1

(a) Simplified representation of the boundless water cycles adapted for fjords of Fjordland National park, NZ (source: pxhere). NP are washed out during various processes and enriched within water reservoirs. Points of sampling are exemplarily represented by white arrows.

(b) Overview of experimental workflow. After taking water samples, the organic material contained in these samples was concentrated and the samples were desalted and cleaned by solid phase extraction (SPE). The samples were thereafter analyzed with FT-ICR-MS for their chemical composition and with an anti-HIV-1 full replication assay and with a comprehensive High-content screening for *antiviral* and cell-modulatory activity. The obtained results were combined and integratively analyzed. This allows to detect bioactivity *(antiviral)* hotspots, a creation of a NP catalogue and eventually the discovery of novel bioactives.


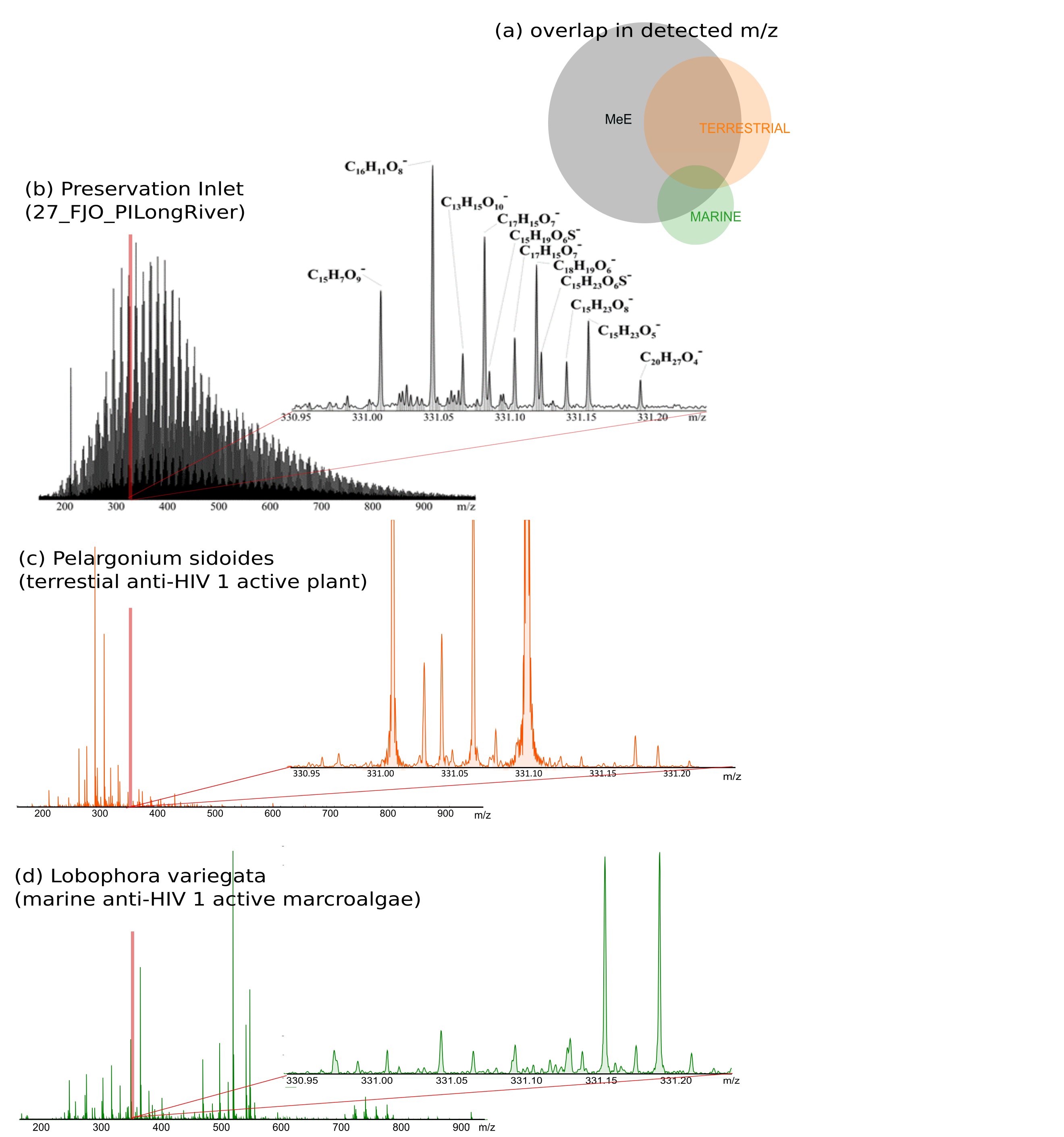


Fig. S2.

Comparison of chemical fingerprints of MeE and two biological extracts, which has been associated with anti-HIV-1 activity, previously ^1,2^. The Venn Diagram (a) gives an overview based on the quantity of detected m/z values, which were converted to elemental compositions: 50% of the detected masses of the marine marcoalgae and 76% of the detected masses of the medical plant pelargonium were present in MeE samples. Natural mixtures like the MeE taken in Preservation inlet (b) are extremely rich and complex compared to biological extracts given in (c) for Pelargonium sidoides and (d) Lobophora variegata. The broad band 12T FT-ICR-MS spectra are illustrated for all three extracts. Furthermore, a small section of the mass spectra (330.95 - 331.20 Da, randomly chosen and highlighted in red in the broad band spectra) are given for all samples.


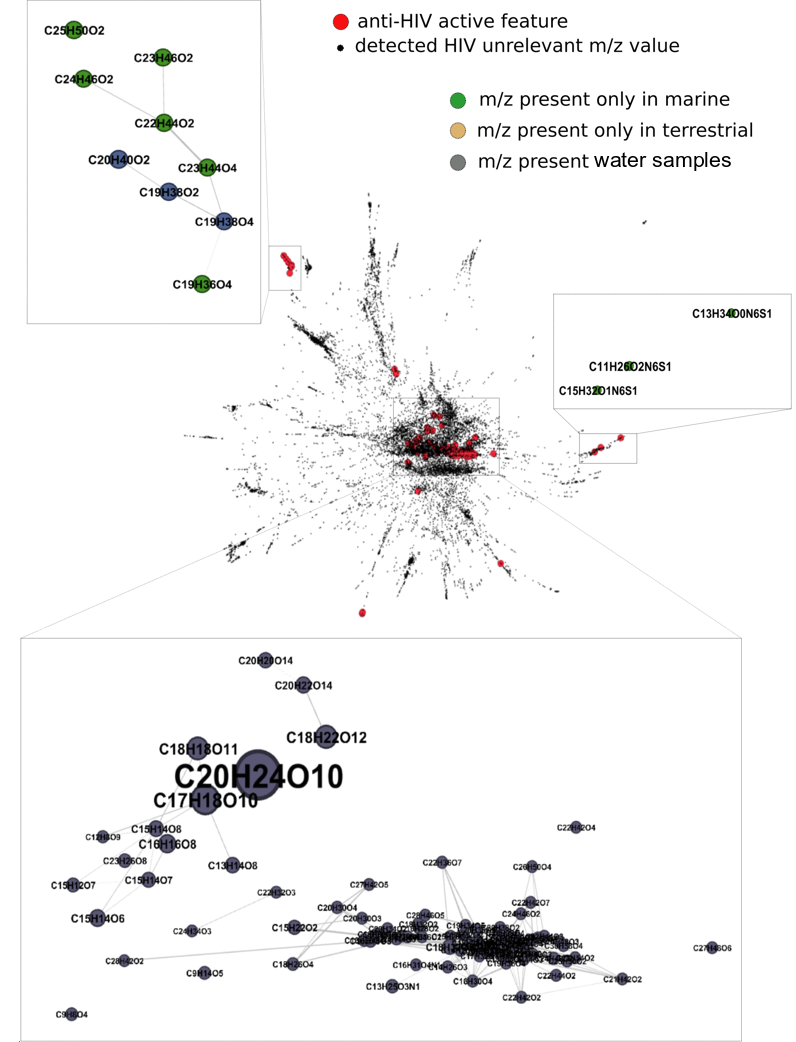


Fig. S3.

Comparison of NPs of two single organisms with pronounced anti-HIV activity, the medical plant Pelargonium sidoides and marine macroalga Lobophora variegata, and the MeE sample set ^1,2^. Remarkably, 94 out of 107 elemental compositions that were previously associated with anti-HIV activity of these organisms were present in the MeE. We performed a mass difference based network analysis of the combined data for Pelargonium sinoides, Lobophora variegate and MeE, which allows data visualization and connection of m/z values by typically appearing chemical transformation reactions ^3,4^. Therefore, it is possible to compare also the presents of closely related elemental compositions like in homologous series. Elemental compositions, which have been associated with anti-HIV activity in previously published work are colored in red and HIV-unrelated features in black in the holistic network. A close clustering of several anti-HIV associated m/z features can be observed. The three main modules containing these m/z features are highlighted in grey boxes and are enlarged illustrated. In these boxes we used a color code according to the detection of the m/z feature in only biological: either in the terrestrial (ochre) or in the marine sample (green), or in biological extract and MeE samples (grey). Node sizes represent here the intensity of the related m/z feature. Within these three main modules no m/z feature has been exclusively present in terrestrial plant Pelargonium sidoides. Even though some are unique in the marine sample, most are present in the MeE, which supports our working hypothesis of MeE being a rich source of NPs.


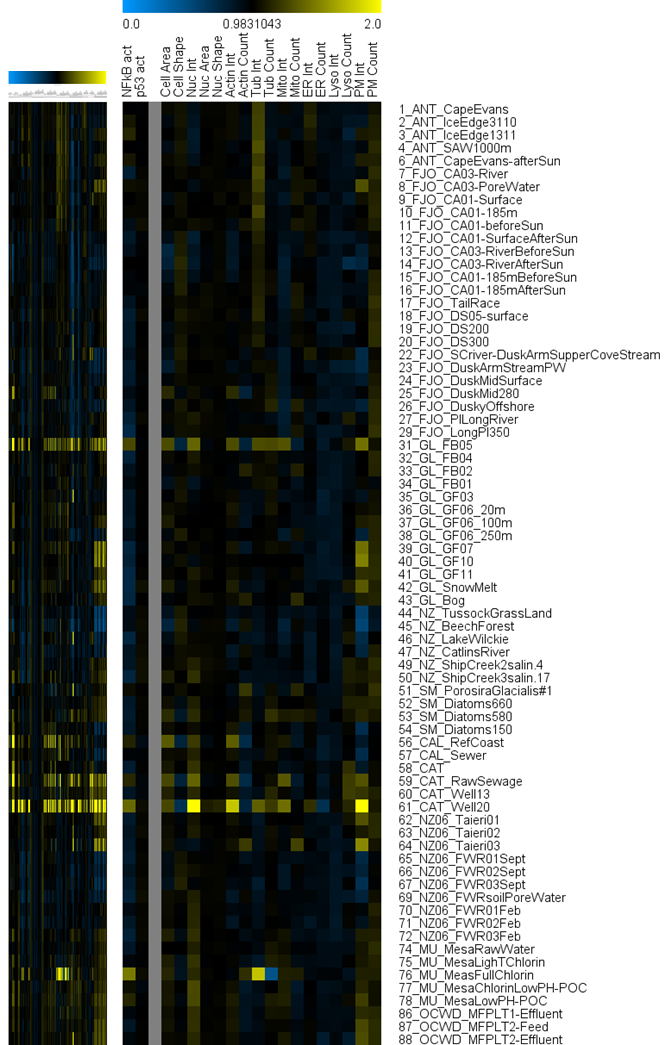


Fig. S4.

Cytological profiles showing a reduced set of 19 core markers are displayed for easier visualization of affected processes and a vertical grey bar was used to separate between regulatory (NFkB, p53) and other cellular markers. Full cytological profiles are shown on the left side. Colors indicate positive (yellow) or negative (blue) deviation from the mean of untreated control cells for each cellular feature (control = 1).


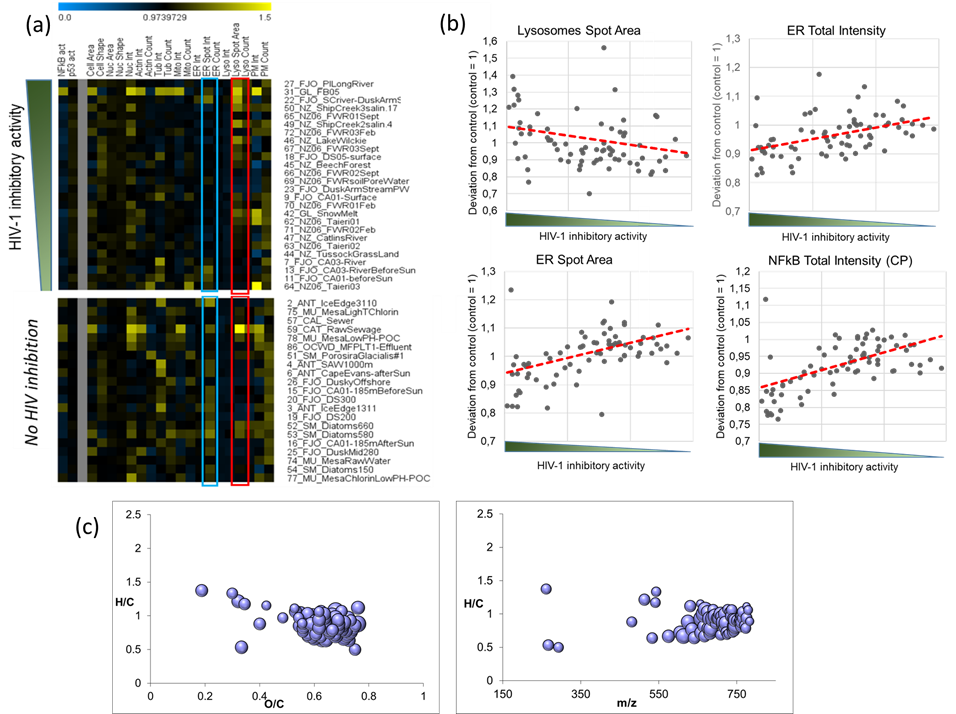


Fig. S5.

Comparison of cytological profiles of HIV-1 inhibitory and inactive samples gives a modulation in ER- and lysosome-related cellulare features. (a) Comparison of selected cytological profiles of HIV-1 inhibitory (>50% inhibition) and inactive samples. Two cellular features (ER spot intensity (blue square) and lysosomes spot area (red square)), that differ considerably between HIV-1 inhibitory and inactive samples are highlighted. A reduced set of 19 core markers are displayed for easier visualization of affected processes and a vertical grey bar was used to separate between regulatory (i.e. NFkB, p53) and other cellular markers. Colors indicate positive (yellow) or negative (blue) deviation from the mean of untreated control cells for each cellular feature (control = 1). (b) Visualization of correlation between two endoplasmic reticulum-related features (ER spot area and ER total intensity), lysosomal (lysosomes spot area) and NFkB-related feature (NFkB total intensity in the cytoplasm) with HIV inhibition. A trendline was added to visualize positive or negative correlations. (c) Van Krevelen diagram illustrating H/C vs O/C and illustration H/C vs m/z for m/z features, that correlated well (correlation koefficent > 0.6) with HIV inhibition over the entire dataset. All of them show specific characteristics of polyphenols (CHO composition, H/C vs O/C, H/C vs m/z).

Table S1.

Easy Hit readout for all tested samples: In the table all samples are listed according to their sampling site. The full replication assay EASY HIT ^5^ uses a stable fluorescent reporter gene activated by HIV Tat and Rev. The HIV replication was calculated based on the observed fluorescents of treated cells compared to the fluorescents of non-treated infected cells. To measure the viability of the cells a MTT (3-(4,5-dimethylthiazol-2-yl)- 2,5-diphenyl tetrazolium bromid) test was performed. It measures the viability due to the metabolism rate of the cells ^5^. The viability of treated cells is illustrated as percentage ration compared to non-treated control cells.

ANT Antarctica, FJO Fjordland National Park, GL Greenland, NZ New Zealand, SM, Sur surface, PW pore water, CAT Catalina Island, CAL California, F max Fluorenzenz aximum, OCWD Orange County Water District, MU Mesa utilities). Details on the routes of cruises and transcients of samplings are given in the main text references ^28,40,41,42,43^.

| Ecosystems worldwide | | |
| --- | --- | --- |
|  | HIV Replication | MTT |
| 1_ANT_CapeEvans | 86% | 94% |
| 2_ANT_IceEdge3110 | 91% | 87% |
| 3_ANT_IceEdge1311 | 117% | 91% |
| 4_ANT_SAW1000m | 100% | 90% |
| 6_ANT_CapeEvans-afterUVstress | 106% | 92% |
| 7_FJO_CA03-River | 41% | 103% |
| 8_FJO_CA03-PoreWater | 52% | 111% |
| 9_FJO_CA01-Surface | 19% | 114% |
| 10_FJO_CA01-185m | 79% | 107% |
| 11_FJO_CA01-beforeUVstress | 48% | 107% |
| 12_FJO_CA01-SurfaceAfterUVstress | 83% | 104% |
| 13_FJO_CA03-RiverBeforeUVstress | 44% | 110% |
| 14_FJO_CA03-RiverAfterUVstress | 67% | 106% |
| 15_FJO_CA01-185mBeforeUVstress | 108% | 104% |
| 16_FJO_CA01-185mAfterUVstress | 124% | 119% |
| 17_FJO_TailRace | 62% | 108% |
| 18_FJO_DS05-surface | 11% | 123% |
| 19_FJO_DS200m | 118% | 115% |
| 20_FJO_DS300m | 110% | 110% |
| 21_FJO_DS380m | 1% | 3% |
| 22_FJO_SCriver-DuskArmSupperCoveStream | 6% | 106% |
| 23_FJO_DuskArmStream-PW | 18% | 108% |
| 24_FJO_DuskMid-Surface | 60% | 103% |
| 25_FJO_DuskMid-280m | 129% | 98% |
| 26_FJO_Dusky-Offshore | 107% | 94% |
| 27_FJO_PI-LongRiver | 2% | 104% |
| 27_FJO_PI-LongSurface | 7% | 107% |
| 29_FJO_LongPI350m | 73% | 102% |
| 30_GL_Black Ice | 95% | 103% |
| 31_GL_FB05 | 5% | 108% |
| 32_GL_FB04 | 61% | 107% |
| 33_GL_FB02 | 71% | 106% |
| 34_GL_FB01 | 88% | 98% |
| 35_GL_GF03 | 78% | 104% |
| 36_GL_GF06_20m | 79% | 101% |
| 37_GL_GF06_100m | 73% | 99% |
| 38_GL_GF06_250m | 84% | 108% |
| 39_GL_GF07 | 64% | 100% |
| 40_GL_GF10 | 61% | 104% |
| 41_GL_GF11 | 76% | 97% |
| 42_GL_SnowMelt | 25% | 109% |
| 43_GL_Bog | 59% | 102% |
| 44_NZ_TussockGrassLand | 39% | 105% |
| 45_NZ_BeechForest | 12% | 108% |
| 46_NZ_LakeWilckie | 10% | 103% |
| 47_NZ_CatlinsRiver | 34% | 104% |
| 48_NZ_ShipCreek1forest | 17% | 112% |
| 49_NZ_ShipCreek2salin.4 | 9% | 114% |
| 50_NZ_ShipCreek3salin.17 | 6% | 119% |
| 51_SM_PorosiraGlacialis#1 | 96% | 106% |
| 52_SM_Diatoms660 | 121% | 117% |
| 53_SM_Diatoms580 | 122% | 110% |
| 54_SM_Diatoms150 | 137% | 102% |
| 62_NZ_Taieri01 | 33% | 97% |
| 63_NZ_Taieri02 | 36% | 101% |
| 64_NZ_Taieri03 | 50% | 104% |
| 65_NZ06_FWR01Sept | 7% | 100% |
| 66_NZ06_FWR02Sept | 15% | 99% |
| 67_NZ06_FWR03Sept | 10% | 105% |
| 68_NZ06_FWR04Sept | 75% | 97% |
| 69_NZ06_FWRsoilPoreWater | 17% | 116% |
| 70_NZ06_FWR01Feb | 19% | 125% |
| 71_NZ06_FWR02Feb | 33% | 122% |
| 72_NZ06_FWR03Feb | 9% | 110% |
| 73_NZ06_FWR04Feb | 55% | 106% |
|  |  |  |
|  |  |  |
| POLARSTERN CRUISE WATER SAMPLES | | |
|  | HIV Inhibition | MTT |
| Rott1 | 73% | 99% |
| Rott2 | 78% | 102% |
| Chan3 | 81% | 101% |
| S_4 3.11. | 82% | 104% |
| S_5 3.11. | 95% | 95% |
| N6 | 83% | 100% |
| D7 | 73% | 100% |
| N8 | 95% | 95% |
| D9 | 82% | 102% |
| N10 | 90% | 102% |
| S_11 | 98% | 106% |
| N12 | 93% | 107% |
| D13 | 79% | 103% |
| N14 | 96% | 91% |
| S_15 | 79% | 100% |
| N16 | 89% | 98% |
| D17 | 80% | 101% |
| N18 | 82% | 105% |
| D19 | 91% | 100% |
| N20 | 92% | 89% |
| D21 | 80% | 100% |
| N22 | 98% | 103% |
| S_23-12 | 86% | 105% |
| N24 | 89% | 107% |
| S_25 | 100% | 104% |
| N26 | 101% | 101% |
| S_27-14 | 102% | 100% |
| N28 | 98% | 89% |
| D29 | 102% | 100% |
| N30 | 97% | 102% |
| Smily | 103% | 99% |
| N32 | 99% | 96% |
| D33 | 95% | 97% |
| N34 | 94% | 98% |
| D35 | 91% | 102% |
| N36 | 94% | 102% |
| D37 | 101% | 101% |
| N38 | 106% | 99% |
| D39 | 101% | 98% |
| N40 | 101% | 94% |
| D41 | 90% | 97% |
| N42 | 91% | 100% |
| D43 | 90% | 101% |
| N44 | 88% | 105% |
| D45 | 95% | 98% |
| N46 | 92% | 98% |
| D47 | 90% | 102% |
| N48 | 90% | 103% |
| D49 | 92% | 101% |
| N50 | 94% | 102% |
| D51 | 96% | 99% |
| N52 | 93% | 100% |
| D53 | 98% | 87% |
| N54 | 94% | 102% |
| D55 | 91% | 101% |
| N56 | 92% | 102% |
| D57 | 95% | 100% |
| N58 | 98% | 98% |
| D59 | 88% | 102% |
| N60 | 92% | 100% |
| D61 | 94% | 106% |
| N62 | 94% | 98% |
| D63 | 94% | 96% |
| N64 | 95% | 99% |
|  |  |  |
| POLARSTERN CRUISE FILTER | | |
|  | HIV Inhibition | MTT |
| SU1 | 68% | 100% |
| ST2 <20 | 94% | 104% |
| ST2>20 | 97% | 103% |
| ST2 >55 | 92% | 102% |
| ST3 A surf | 76% | 104% |
| ST5 <20 | 87% | 98% |
| ST3 B 400m | 78% | 97% |
| ST3 C 1500m | 56% | 93% |
| ST3 D 2500m | 71% | 87% |
| ST3 E <0,2-4700m | 79% | 100% |
| ST4 A surf | 86% | 105% |
| ST5 surf | 85% | 103% |
| ST5 >20 | 90% | 106% |
| ST5 >55 | 87% | 105% |
| ST6 | 89% | 97% |
| ST7 A surf | 98% | 99% |
| ST7 B 500m | 72% | 94% |
| ST7 C 1100m | 79% | 93% |
| ST7 D 4022m | 73% | 84% |
| ST8 surf | 71% | 92% |
| ST9 surf A | 72% | 91% |
| ST9 B <20 | 51% | 84% |
| ST9 >20 | 92% | 103% |
| ST9 >55 | 83% | 102% |
| ST10 surf C | 72% | 87% |
| ST11 surf A | 86% | 95% |
| ST11 <20 | 59% | 92% |
| ST11>20 | 95% | 104% |
| ST11>55 | 92% | 103% |
| ST11 B 89m | 74% | 97% |
| ST11 D 1300m | 51% | 89% |
| ST11 C 4123m | 51% | 88% |
| ST12 surf | 64% | 94% |
| ST13 surf | 77% | 95% |
| ST13<20 | 45% | 87% |
| ST13>20 | 55% | 102% |
| ST13>55 | 93% | 103% |
| ST14 surf 1 | 72% | 89% |
| ST14 B 49m | 80% | 94% |
| ST14 C 1600m | 96% | 98% |
| ST14 D 4802m | 71% | 93% |
| ST15 surf | 71% | 91% |
| ST16 | 69% | 92% |
| ST16<20 | 77% | 92% |
| ST16>20 | 91% | 99% |
| ST16>55 | 89% | 100% |
| ST17 surf | 90% | 99% |
| ST18 | 79% | 97% |
| ST19 | 73% | 93% |
| ST19<20 | 102% | 101% |
| ST19>20 | 99% | 104% |
| ST19>55 | 99% | 102% |
| ST20 surf | 75% | 94% |
| ST20-62m Fmax | 75% | 94% |
| ST20-1800m | 74% | 95% |
| ST20-4840m | 71% | 93% |
| ST21 surf | 79% | 96% |
| ST22 | 84% | 97% |
| ST22 <20 | 75% | 95% |
| ST22 >20 | 92% | 102% |
| ST22 >55 | 93% | 101% |
| ST23 | 99% | 99% |
| ST23 <20 | 76% | 94% |
| ST23 >22 | 101% | 105% |
| ST23 >55 | 101% | 103% |
| ST24 surf | 84% | 99% |
| ST24-51m | 83% | 98% |
| ST24-5461m | 82% | 95% |
| ST25 | 89% | 92% |
| ST25-1797m | 91% | 84% |
| ST26A surf | 86% | 94% |
| ST26C-4600m | 75% | 91% |
| ST26B-1800m | 72% | 88% |
|  |  |  |
|  |  |  |
| POLARSTERN CRUISE BURST | | |
|  | HIV Inhibition | MTT |
| W3 | 72% | 91% |
| W4 | 75% | 89% |
| W5 | 88% | 89% |
| W6 | 95% | 100% |
| W7 | 95% | 100% |
| W8 | 80% | 91% |
| W9 | 85% | 95% |
| W10 | 93% | 95% |
| W11 | 79% | 100% |
| W12 | 74% | 102% |
| W13 | 94% | 97% |
| W14 | 87% | 102% |
| W15 | 94% | 105% |
| W16 | 93% | 99% |
| W17 | 85% | 100% |
| W18 | 93% | 101% |
| W19 | 87% | 97% |
| W20 | 91% | 101% |
| W21 | 92% | 103% |
| W22 | 101% | 88% |
| W23 | 88% | 101% |
| W24 | 78% | 100% |
| W25 | 66% | 104% |
| W26 | 95% | 97% |
| W27 | 92% | 105% |
| B4 | 95% | 100% |
| B5 | 91% | 97% |
| B6 | 83% | 99% |
| B7 | 90% | 102% |
| B8 | 87% | 106% |
| B9 | 90% | 101% |
| B10 | 89% | 104% |
| B11 | 57% | 84% |
| B12 | 81% | 95% |
| B13 | 93% | 95% |
| B14 | 93% | 105% |
| B15 | 95% | 104% |
| B16 | 97% | 103% |
| B17 | 81% | 108% |
| B18 | 96% | 103% |
| B19 | 85% | 104% |
| B20 | 89% | 103% |
| B22 | 90% | 101% |
| B23 | 94% | 98% |
| B24 | 74% | 90% |
| B25 | 76% | 103% |
| B26 | 94% | 98% |
| B27 | 86% | 105% |
| B28 | 91% | 103% |
| B29 | 88% | 102% |
| B30 | 86% | 106% |
| C5 | 92% | 100% |
| C6 | 87% | 100% |
| C7 | 69% | 104% |
| C8 | 85% | 102% |
| C9 | 85% | 102% |
| C10 | 88% | 97% |
| C11 | 85% | 99% |
| C12 | 91% | 98% |
| C13 | 83% | 102% |
| C14 | 81% | 100% |
| C15 | 85% | 104% |
| C16 | 78% | 101% |
| C17 | 76% | 78% |
| C18 | 75% | 99% |
| C19 | 85% | 102% |
| C20 | 79% | 100% |
| C22 | 74% | 98% |
| C23 | 89% | 99% |
| C25 | 87% | 101% |
| C26 | 89% | 99% |
| C27 | 94% | 98% |
| C28 | 86% | 98% |
| C29 | 93% | 97% |
| C30 | 91% | 99% |
| D4 | 90% | 100% |
| D5 | 90% | 97% |
| D9 | 72% | 105% |
| D14 | 76% | 101% |
| D17 | 88% | 99% |
| Outflow 9 | 84% | 100% |
| Fmax 28 | 89% | 99% |
| S28 | 67% | 94% |
|  |  |  |
| Ecological samples taken from man-derived systems | | |
|  | HIV Inhibition | MTT |
| 56_CAL_RefCoast | 85% | 100% |
| 57_CAL_Sewer | 95% | 102% |
| 58_CAT | 88% | 97% |
| 59_CAT_RawSewage | 95% | 112% |
| 60_CAT_Well13 | 79% | 118% |
| 61_CAT_Well20 | 55% | 102% |
| 74_MU_MesaRawWater | 129% | 101% |
| 75_MU_MesaLighTChlorin | 92% | 107% |
| 76_MU_MeasFullChlorin | 77% | 111% |
| 77_MU_MesaChlorinLowPH-POC | 146% | 109% |
| 78_MU_MesaLowPH-POC | 95% | 113% |
| 79_OCWD_UVP21.1.09 | 182% | 116% |
| 80_OCWD_ROP21.1.09 | 167% | 122% |
| 81_OCWD_MFPLT 1 Feed | 157% | 119% |
| 86_OCWD_MFPLT1-Effluent | 95% | 100% |
| 87_OCWD_MFPLT2-Feed | 83% | 105% |
| 88_OCWD_MFPLT2-Effluent | 84% | 100% |
| 92_OCWD MFE 20.08.09 | 67% | 113% |
| 93_OCWD MFE 19.09.08 | 57% | 106% |

**Table S2 –** MassTRIX output for the annotation of one of 10 VIP m/z features

| ID | raw mass | exact mass (adduct) | error ppm | formula | database_mass | name |
| --- | --- | --- | --- | --- | --- | --- |
| [LMPK12112212](http://www.lipidmaps.org/data/get_lm_lipids_dbgif.php?LM_ID=LMPK12112212) | 585.08855 | 585.088593532059 | -0.07 | C27H22O15 | 586.09586999894 | Quercetin 3-(2''-galloyl-alpha-L-arabinopyranoside) [Flavones and Flavonols [PK1211]] ([M-H]-) |

**Table S3** – Fragmentation pattern for VIP masses, which could be isolated for SORI-CID

| **m/z** | **FORMULAE** | **Neutral loss** | | | | **Fragment ion** | | | |
| --- | --- | --- | --- | --- | --- | --- | --- | --- | --- |
|  |  | **H_2_O** | **CH_3_OH** | **CO_2_** | **CH_3_OH + CO_2_** | **1** | **2** | **3** | **4** |
| 529.062 | C24H28O14 | 18.010 | 32.026 | 43.990 | 76.016 | 511 | 497 | 485 | 453 |
| 571.073 | C26H20O15 | - | 32.005 | 44.026 | - | 539 | 527 |  |  |

**Supplementary material references**

1 Kremb, S., Muller, C., Schmitt-Kopplin, P. & Voolstra, C. R. Bioactive Potential of Marine Macroalgae from the Central Red Sea (Saudi Arabia) Assessed by High-Throughput Imaging-Based Phenotypic Profiling. *Marine drugs* **15**, doi:10.3390/md15030080 (2017).

2 Helfer, M. *et al.* The root extract of the medicinal plant Pelargonium sidoides is a potent HIV-1 attachment inhibitor. *PLoS One* **9**, e87487, doi:10.1371/journal.pone.0087487 (2014).

3 Moritz, F., Kaling, M., Schnitzler, J. P. & Schmitt-Kopplin, P. Characterization of poplar metabotypes via mass difference enrichment analysis. *Plant Cell Environ* **40**, 1057-1073, doi:10.1111/pce.12878 (2017).

4 Tziotis, D., Hertkorn, N. & Schmitt-Kopplin, P. Kendrick-analogous network visualisation of ion cyclotron resonance Fourier transform mass spectra: improved options for the assignment of elemental compositions and the classification of organic molecular complexity. *Eur J Mass Spectrom (Chichester)* **17**, 415-421, doi:10.1255/ejms.1135 (2011).

5 Kremb, S. *et al.* EASY-HIT: HIV full-replication technology for broad discovery of multiple classes of HIV inhibitors. *Antimicrob Agents Chemother* **54**, 5257-5268, doi:10.1128/AAC.00515-10 (2010).
